# PROTOCOL: Assessment of publication time in Campbell systematic reviews: A cross‐sectional survey

**DOI:** 10.1002/cl2.1303

**Published:** 2023-01-10

**Authors:** Bei Pan, Long Ge, Zhipeng Wei, Liangying Hou, Honghao Lai, Kehu Yang

**Affiliations:** ^1^ School of Basic Medical Sciences, Evidence‐Based Medicine Centre Lanzhou University Lanzhou China; ^2^ School of Public Health, Evidence‐Based Social Science Research Center Lanzhou University Lanzhou China

## Abstract

This is the protocol for a Campbell systematic review. The objectives are as follows. This study has three main objectives: (1) To examine the time duration from title registration to publication of the protocol for a Campbell systematic review and publication of the completed Campbell systematic review; (2) To describe publication times in accordance with the characteristics of the reviews, which include year of publication, type of review, number of authors, number of collaborative institutions, the time gap between the date the search was conducted and review publication, and the length and complexity of the included review (including the number of pages, the number of tables and figures, the number of studies included in the review, the number and type of analyses undertaken, and the number of references); (3) To describe the differences in publication times between Campbell Review Groups.

## 1 BACKGROUND

### 1.1 Description of the problem or issue

Systematic reviews assemble the best available studies on a specific topic and synthesize the results of potentially eligible studies that can provide more valuable references for policymaking and practice than individual studies (Campbell Collaboration, 2018). Therefore, they are considered to be the highest evidence in evidence classification (Gates & March, 2016). In recent years, systematic reviews have increased in popularity, and since 1991, the number of annual publications has increased by more than 2000% (Ioannidis, 2016).

The Campbell Collaboration (2020) supports the preparation and dissemination of high‐quality systematic reviews to investigate the effectiveness of social programs, policies, and practices, thereby enabling policymakers, practitioners, and the public to make better‐informed decisions about policy interventions (Welch, 2018). There are 12 Campbell Review Groups that focus on specific topics and provide editorial and methodological support to assist authors of the Campbell Collaboration to meet their standards of methodology and reporting. Previous studies have shown that review protocols can reduce interim decision making and bias in the review process (Moher et al., 2015; Shamseer et al., 2015). Once the proposed title is approved and registered, the authors of Campbell Collaboration should submit a review protocol to specify the background, objectives, methods, a priori hypotheses, and statistical analysis for a final review, which is part of the rigorous methodology for the production of a Campbell systematic review (The Campbell Collaboration, 2020). Campbell anticipated that the conduct of a protocol would take half a year, and the final review would take 18 months. The policies and guidelines of Campbell systematic reviews state that review teams are expected to submit a draft protocol to the editor or managing editor of the sponsoring coordinating group no later than one year after approval of the title; once the protocol is approved, it is also advisable for review teams to update the coordinating group editor or managing editor on their progress at least every six months and report any problems that may impede the timely delivery of the draft review (The Campbell Collaboration, 2020). However, the guidelines do not address the time gap to submit the full review, instead they only state that a protocol that has not resulted in a final full review within two years can be withdrawn, with the review topic then being made available to other interested review teams (The Campbell Collaboration, 2020). Despite above guideline, the timeline from title registration to publication of the protocol for a Campbell systematic review and then to the publication of the review itself is still uncertain.

Similar to the Campbell systematic review, Cochrane systematic reviews also have high methodological standards and rigorous publication processes in medicine. Writing a Cochrane protocol will take 2–6 months, while writing a full review could take 1–2 years (Cochrane Community, 2021). Several studies have assessed the publication duration and process of the Cochrane systematic reviews and have shown that complete Cochrane systematic reviews tend to take longer than other systematic reviews (Andersen et al., 2020; Runjic et al., 2019; Tricco et al., 2008).

### 1.2 Why it is important to conduct this review

No studies have examined the time from title registration and publication of the protocol for a Campbell systematic review to the publication of the review itself, and the extent to which Campbell reviews' publication time adheres to current guidelines is unclear. Delayed publication of systematic reviews increases the risk of outdated data. Shojania et al. (2007) found that 7% of systematic reviews had signs of being outdated when published, and 11% showed signs of being outdated within two years of the completion of their systematic search. Wang et al. (2021) performed a systematic review to assess the methodological and reporting quality of Campbell systematic reviews and found that the overall quality was high, especially after the introduction of the Methodological Expectations of Campbell Collaboration Intervention Reviews (MECCIR) in 2014. This may be attributed to the high methodological standards that Campbell reviews must adhere to, which results in longer publication times. The assessment of the publication process and duration of Campbell systematic reviews will prompt them to devise strategies for optimizing and lowering the processing time from writing to publication without compromising on quality. This will reduce the risk of outdated evidence and the time commitment required for publishing a Campbell review.

## 2 OBJECTIVES

This study has three main objectives:

- To examine the time duration from title registration to publication of the protocol for a Campbell systematic review and publication of the completed Campbell systematic review.
- To describe publication times in accordance with the characteristics of the reviews, which include year of publication, type of review, number of authors, number of collaborative institutions, the time gap between the date the search was conducted and review publication, and the length and complexity of the included review (including the number of pages, the number of tables and figures, the number of studies included in the review, the number and type of analyses undertaken, and the number of references).
- To describe the differences in publication times between Campbell Review Groups.

## 3 METHODOLOGY

### 3.1 Eligibility criteria

All Campbell systematic reviews in their first published version will be included. Reviews from each of the following groups will be included: business and management, children and young person's well‐being, climate solutions, crime and justice, disability, education, international development, knowledge translation and implementation, methods, and social welfare. We will exclude reviews (1) if they are duplicates; (2) if they miss the publication date for title registration, protocol, or review; and (3) if they have their protocol amended or updated before the full review gets published.

### 3.2 Electronic searches

We will search the Campbell systematic reviews journals on the Wiley Online Library website to identify all complete studies to date. We will manually search table of contents of all Campbell systematic reviews to obtain the date of title registration information on the website of this journal.

### 3.3 Study screening and selection

Two reviewers will independently screen all Campbell systematic reviews according to the eligibility criteria. Reviewers will resolve conflicts through discussion and adjudication by a third reviewer when necessary.

### 3.4 Data extraction

Two coders will independently code all eligible studies, and any disagreements will be resolved by discussion or a third party. A preliminary version of the coding scheme to be used is presented in Table 1. We will extract title registration dates, protocol publication dates, full review publication dates, types of review, groups of review, topics of review, and number of authors from each of the publicly available records of the review in Campbell systematic reviews journals for both reviews and protocols, number of collaborative institutions, the time gap between the date the search was conducted and review publication, and the length and complexity of the included review (including the number of pages, number of tables and figures, and number of references, the number of studies included in the review, and the number and type of analyses undertaken, and references). The study size will be based on available data from the Campbell Library that meets the eligibility criteria.

**Table 1:** Code

Category	Answer
Basic information	
Title	Open answer
Authors	Open answer
Country	Open answer
Number of Authors	Open answer
Authors' Institutions	Open answer
Publication time	
Title registration dates	Open answer
Protocol publications dates	Open answer
Full review publication dates	Open answer
Characteristics of the reviews	
Campbell coordinating group	Aging
	Business and management
	children and young persons
	Climate solutions
	Crime and justice
	Disability
	Education
	International development
	Knowledge translation & implementation
	Methods
	Social Welfare
Type of review	Systematic review
	Evidence and gap maps
	Methods research paper
Topics of review	Open answer
Time gap between the date the search was conducted and review publication	Open answer
Number of collaborative institutions	Open answer
Length and complexity of included review	Number of pages
	Number of tables
	Number of figures
	Number of references

### 3.5 Data synthesis

We will use the SPSS software to perform the statistical analysis and use descriptive statistics to report publication times, which would be calculated stratified by characteristics, including year of review publication, type, number of authors, type of review, number of collaborative institutions, the time gap between the date the search was conducted and review publication, the length and complexity of the included reviews (including number of pages, number of tables and figures, and number of references), and Campbell Review Groups. Non‐normally distributed data will be reported as medians, interquartile range, and range; whereas, normally distributed data will be reported as mean ± standard deviation. Additionally, we will also visualize the overall publication time and distribution of the data.

## CONTRIBUTIONS OF AUTHORS

Please give brief description of content and methodological expertise within the review team. The recommended optimal review team composition includes at least one person on the review team who has content expertise, at least one person who has methodological expertise and at least one person who has statistical expertise. It is also recommended to have one person with information retrieval expertise.

Who is responsible for the below areas? Please list their names:

- Content: Professor Yang is a professor who has been working in the field of evidence‐based medicine for over 14 years at Evidence Based Medicine Center, School of Basic Medical Sciences, Lanzhou University. Long Ge has an extensive experience in evidence‐based health decision research, policy advocacy, training and teaching at Evidence Based Social Science Research Center, School of Public Health, Lanzhou University.
- Systematic review methods: Kehu Yang and Long Ge have previous experience in systematic review methodology, including searching, data collection, and theory‐based synthesis.
- Statistical analysis: Bei Pan, Liangying Hou, and Zhipeng Wei.
- Information retrieval: Information retrieval will be done by experienced teams at Evidence Based Social Science Research Center, School of Public Health, Lanzhou University.

## POTENTIAL CONFLICTS OF INTEREST

There are no conflicts of interest for this study.

## PRELIMINARY TIMEFRAME

Approximate date for submission of the systematic review.

Date you plan to submit a draft protocol: June 2022.

Date you plan to submit a draft review: August 2023.

## PLANS FOR UPDATING THIS REVIEW

Note: Please specify how the review will be updated. This should include, at a minimum, information on who will be responsible and the frequency with which updates can be expected.

## SOURCES OF SUPPORT

**Internal sources**

- Major Project of the National Social Science Fund of China, China

‘Research on the Theoretical System, International Experience and Chinese Path of Evidence‐based Social Science’ (Project No. 19ZDA142).

**External sources**

- NONE, Other
